# Effect of Pollen Storage Duration on Stainability, Fruit Set, and Physical Traits in Date Palm (*Phoenix dactylifera* L.) Cultivar ‘Mejhoul’

**DOI:** 10.3390/plants14203189

**Published:** 2025-10-17

**Authors:** Ricardo Salomón-Torres, Mohammed Aziz Elhoumaizi, Carlos Zambrano-Reyes, Abdelouahhab Alboukhari Zaid, Yohandri Ruisanchez-Ortega, Laura Patricia Peña-Yam, María Melissa Gutiérrez-Pacheco

**Affiliations:** 1 Unidad Académica San Luis Río Colorado, Universidad Estatal de Sonora, San Luis Río Colorado C.P. 83500, Sonora, Mexico; yohandri.ruisanchez@ues.mx (Y.R.-O.); laura.penayam@ues.mx (L.P.P.-Y.); 2 Laboratory for Agricultural Productions Improvement, Biotechnology and Environment (LAPABE), Faculty Sciences, University Mohammed First, BP-717, Oujda 60000, Morocco; 3 Jefatura de Distrito 002 Río Colorado, Oficina de Representación en Baja California de la SADER, Mexicali C.P. 21100, Baja California, Mexico; carlos.zambranor@bc.agricultura.gob.mx; 4 Khalifa International Award for Date Palm and Agricultural Innovation, Abu Dhabi P.O. Box 3614, United Arab Emirates; abdelouahhabz@diwan.gov.ae; 5 Departamento de Ingenierías, Tecnológico Nacional de México/Instituto Tecnológico del Valle del Yaqui, Bácum C.P. 85276, Sonora, Mexico; maria.gp@vyaqui.tecnm.mx

**Keywords:** date palm, *Phoenix dactylifera* L., ‘Mejhoul’, pollen storage, acetocarmine stainability, fruit set, metaxenia

## Abstract

Asynchronous flowering between male and female date palms (*Phoenix dactylifera* L.) makes pollen storage a practical necessity for growers, especially for cultivars like ‘Mejhoul’, which require artificial pollination. This study examined the stainability of pollen as an indicator of cytoplasmic integrity, from four male date plant pollen donor genotypes (‘Mejhoul’, ‘Deglet Nour’, ‘Khadrawy’, and ‘Zahidi’) stored at 4 °C for different durations (fresh, one-year, and two-year storage) and their effects on fruit set and physical fruit characteristics of the Mejhoul cultivar in Mexico. Pollen stainability was assessed in vitro using 1% acetocarmine. Fruit and seed set percentages were evaluated as indicators of the practical effectiveness of stored pollen under field conditions, but not as direct measures of viability. Results showed that fresh pollen exhibited the highest stainability (91.2–95.6%), followed by one-year-stored pollen (59.4–68.3%), and two-year-stored pollen (38.8–45.4%). Fruit set percentages were highest with fresh pollen (63.8–81.7%), decreasing with storage duration. ‘Deglet Nour’ pollen consistently showed superior compatibility with ‘Mejhoul’ females. Physical fruit characteristics (weight, length, diameter) and seed traits were minimally affected by reduced pollen stainability, indicating that there were enough viable grains for effective pollination. The study also observed Metaxenia and Xenia effects, where pollen genotypes influenced fruit and seed size. Overall, these findings suggest that pollen stored at 4 °C for short and medium terms can be used in Mejhoul production, but longer storage significantly reduces efficacy, recommending sub-zero temperatures for extended preservation.

## 1. Introduction

Pollen viability is a critical factor in plant reproduction, particularly in agriculturally important species such as the date palm (*Phoenix dactylifera* L.), where effective pollination is vital for ensuring high-quality fruit production [1]. As a dioecious species, date palms rely on cross-pollination to achieve adequate fruit set and quality, making pollen viability a key factor of successful artificial pollination [2,3]. Viability is a term that is often inferred from its evaluation by means of germination tests, where cytological staining remains a useful indirect indicator to evaluate the condition of pollen in the context of its cytoplasmic integrity.

One of the main challenges in date palm production is ensuring the availability of viable pollen during the female flowering period, particularly when male and female flowers exhibit asynchronous maturation [3]. This has led growers to rely on pollen storage for later use, necessitating an evaluation of how storage temperature and duration affect pollen viability [4]. In this context, pollen storage plays a crucial role in preserving viability and germination capacity over necessary periods by optimizing preservation conditions.

Maintaining pollen viability during storage is crucial for optimizing artificial pollination processes, particularly in regions with limited access to fresh pollen or where long-distance transport is required [2]. However, pollen viability can be affected by factors such as storage temperature and environmental exposure time, underscoring the need to establish optimal preservation conditions [5].

Recent studies have evaluated a wide range of storage conditions to extend pollen shelf life and facilitate artificial pollination, including refrigeration at 4 °C in a desiccator, freezing at −20 °C, and cryopreservation at −196 °C (liquid nitrogen). Cryopreservation was found to be the most effective for long-term preservation, maintaining viability levels between 75% and 84% [6]. Similarly, standard protocols in germplasm banks, such as those established by the USDA, report that pollen can remain viable for at least nine months in liquid nitrogen without significant loss and can withstand multiple freeze–thaw cycles [7].

Pollen viability varies across genotypes and storage methods, as the collection stage (floral stage), initial moisture content, and pre-storage drying protocols significantly influence outcomes [8]. For most growers, storage at −20 °C or refrigeration at 4 °C, combined with proper drying and sealed containers, are the most accessible and effective methods for maintaining viability for several months or up to a year, with temperature and relative humidity being the most critical determinants [6,9].

In commercial plantations, the female inflorescence is pollinated with pollen from selected males. Date growers are aware that pollen from different sources can have an immediate impact on the properties of seeds and fruits of the date palm. Pollen from different date sources can affect ripening time, color, weight, size, and other qualities of the date palm fruit [10,11]. The effect of pollen on the fruit is a phenomenon known as metaxenia [12] and on the seed as xenia [13]. This was initially described for *Phoenix dactylifera*, but similar effects have also been reported in other crops such as maize, grapes, and in cucumber [14,15,16].

In Mexico, studies have focused on the effects of different pollen sources on the chemical, nutritional, and quality characteristics of ‘Mejhoul’ dates [17,18]. However, no research has investigated the stainability and effects of stored pollen across different storage periods on the fruit of the ‘Mejhoul’ cultivar.

The objectives of this study were: (1) to evaluate the stainability of fresh date palm pollen compared to pollen stored at 4 °C for one and two years, as an indirect estimate of viability; (2) to compare the effects of four pollen genotypes on the female ‘Mejhoul’ cultivar grown in Mexico; and (3) to analyze the influence of pollination with pollen stored for up to two years at 4 °C on the physical properties of the fruit.

## 2. Results and Discussion

### 2.1. Pollen Stainability Results

**Figure 1:**
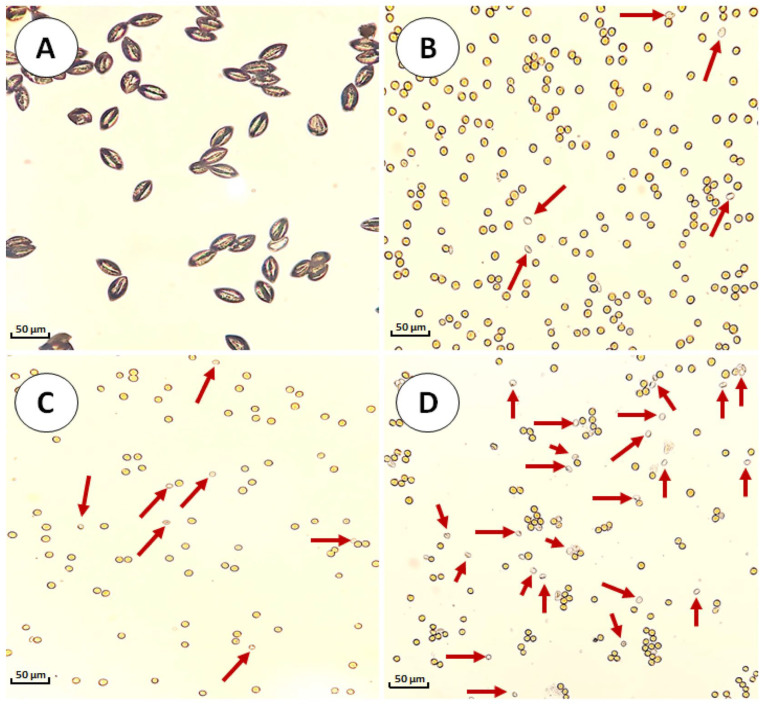
Stainability of date palm pollen stored at 4 °C assessed via acetocarmine staining. Intensely stained grains are classified as acetocarmine-positive, indicating preserved cytoplasmic integrity, whereas partially stained or colorless grains are considered acetocarmine-negative. Differences in pollen morphology (shrunken vs. round) are due to the hydration state of the samples: Image (**A**) was taken from dry pollen, while images (**B**–**D**) show hydrated pollen stained with acetocarmine. (**A**) Mature unstained pollen grains (40×). (**B**) Fresh pollen (60×). (**C**) One-year-stored pollen (60×). (**D**) Two-year-stored pollen (60×).

Pollen stainability was evaluated using acetocarmine staining, which distinguishes acetocarmine-positive (stained) from acetocarmine-negative (unstained) pollen grains based on cytoplasmic integrity rather than functional viability. Unlike germination tests, which assess functional viability through pollen tube emergence, acetocarmine staining only reveals the presence of intact cytoplasmic components and serves as an indirect structural indicator of pollen condition. Figure 1A shows unstained pollen grains with characteristic ellipsoidal morphology and intact outer walls, providing a baseline for comparison with treated samples. Figure 1B–D illustrate acetocarmine-stained fresh pollen, and pollen stored for one and two years at 4 °C, respectively.

Microscopic examination estimated the proportion of acetocarmine-positive (intensely stained) and acetocarmine-negative (partially stained, weakly stained, or unstained, indicated with red arrows in Figure 1B–D) pollen grains. Staining revealed notable differences in coloration intensity among samples. Figure 1B, representing fresh pollen, showed a high proportion of acetocarmine-positive grains (91.2–95.6%), reflecting high cytoplasmic integrity at the time of collection. These results are consistent with previous studies reporting 92–96% acetocarmine-positive pollen for freshly collected *Phoenix dactylifera* pollen using similar staining methods [19,20,21].

In contrast, Figure 1C, representing one-year-stored pollen, showed a reduction in acetocarmine-positive grains (59.4–68.3%), suggesting partial loss of cytoplasmic integrity due to storage, though such pollen remained potentially functional for pollination. This pattern agrees with studies reporting acceptable levels de acetocarmine-grains (62–63%) after 12 months of storage [19,21]. Figure 1D, representing two-year-stored pollen, showed a further decrease in acetocarmine-positive grains (38.8–45.4%), reflecting significant deterioration of cellular integrity after prolonged storage. This progressive loss suggests that extended storage negatively affects pollen cytoplasmic cellular integrity, as suggested by the results described in Table 1. It is worth noting that, to our knowledge, no previous studies have evaluated date palm pollen stainability after two years of storage at 4 °C.

Acetocarmine staining is a commonly used technique for date pollen research. It is rapid, inexpensive, and allows viable pollen with intact cytoplasm to be distinguished thanks to its bright red color [22]. This method is simple and accessible, allowing date palm growers to analyze large numbers of samples [23], without requiring sophisticated laboratory technology and processes for in vivo testing.

### 2.2. Fruit Set Percentages

Table 2 presents the fruit set percentages obtained with pollen from four date palm pollen donor sources (MJL, KDY, DGN, and ZHI) under three storage durations across two growing seasons. Pollen treatments included freshly collected pollen (current season, 2022 and 2023), one-year-stored pollen (collected in 2021 and 2022 for the 2022 and 2023 seasons, respectively), and two-year-stored pollen (collected in 2020 and 2021 for the 2022 and 2023 seasons, respectively).

Fresh pollen (2022 and 2023), one-year-stored pollen (2021 and 2022), and two-year-stored pollen (2020 and 2021) from the DGN donor exhibited the highest fruit set percentages, suggesting high compatibility with female ‘Mejhoul’ plants [24]. MJL pollen had the lowest fruit set percentages with two-year-stored pollen but remained competitive with fresh and one-year-stored pollen in both seasons. ZHI fresh pollen (2022) and one-year-stored pollen (2021 and 2022) showed lower fruit set percentages than other donors, except for KDY fresh pollen in 2023, which was lower than other donors that year. This suggests that ZHI pollen may have lower performance in ‘Mejhoul’ females. All two-year-stored pollen sources exhibited the lowest fruit set percentages overall, with a notable decrease in efficacy. Kadri, et al. (2022) [19] reported fruit set percentages of 89.66% for fresh pollen and 63.02% for one-year-stored pollen at 4 °C in the ‘Deglet Nour’ cultivar. In this study, MJL averaged 70.08% for fresh pollen and 57.66% for one-year-stored pollen across both seasons. Differences between studies may stem from the use of pure versus diluted pollen and the different cultivars tested.

Table 3 provides a quantitative analysis of the percentage reduction in fruit set between fresh and stored pollen, comparing the MJL, KDY, DGN, and ZHI donors under different storage conditions.

The average decrease in fruit set percentage between fresh and one-year-stored pollen ranged from 19.53–23.07%, while the decrease between fresh and two-year-stored pollen ranged from 36.65–52.41%. The overall average decrease was 21.45% for one-year-stored pollen and 46.12% for two-year-stored pollen, highlighting the loss of pollen viability with prolonged storage.

### 2.3. Fruit and Seed Characteristics

Table 4 shows that fruit weight varied by pollen source and storage condition. In 2022, fresh DGN pollen produced significantly heavier fruits (21.18 g) compared to MJL (18.05 g), while in 2023, fresh KDY pollen yielded the heaviest fruits (22.70 g). One- and two-year-stored pollen maintained comparable performance, with weights ranging from 18.47 to 22.47 g, showing no significant differences in most cases. Pollen stainability, decreasing from 91–95% (fresh) to 59–68% (one year) and 38–45% (two years), did not correlate directly with significant reductions in fruit weight. For fruit length, fresh pollen in 2022 produced significant differences, with ZHI (4.56 cm) and DGN (4.49 cm) yielding longer fruits than MJL (4.10 cm). In 2023, differences among donors diminished, with lengths ranging from 4.16 to 4.69 cm for fresh pollen. For stored pollen, lengths were similar, with DGN in 2022 (4.77 cm) and all donors in 2021 (4.97–5.17 cm) producing slightly longer fruits. Fruit diameter showed less variability; in 2022, fresh ZHI and DGN pollen produced larger diameters (2.69 and 2.62 cm, respectively) compared to MJL (2.42 cm). In 2023, values were more uniform (2.30–2.59 cm). For stored pollen, diameters ranged from 2.40–2.67 cm, with no significant differences in most cases.

These results suggest that, despite lower stainability in stored pollen, the quantity of acetocarmine-positive grains remains sufficient to ensure effective pollination and adequate fruit development in the ‘Mejhoul’ cultivar. However, since staining does not necessarily imply germinability, observed fruit set should be interpreted as an indirect reflection of field performance rather than a direct measure of pollen viability. The reduced stainability of one- and two-year-stored pollen did not significantly limit fruit size, diameter, or length, indicating that, although cytoplasmic integrity declined, the pollen retained sufficient apparent functional performance for successful fertilization under field conditions.

Differences observed among pollen donors, particularly with DGN and ZHI producing heavier and longer fruits in some cases, suggest a Metaxenia effect, where the pollen genotype influences fruit characteristics [12]. This phenomenon appears to occur independently of pollen stainability, as both fresh and stored pollen from these donors yield similar results. These findings highlight the importance of selecting specific pollen sources to optimize fruit quality in the ‘Mejhoul’ cultivar [3,24].

Table 5 shows that seed weight varied by pollen donor and storage condition. In 2022, fresh MJL (1.47 g) and ZHI (1.43 g) pollen produced significantly heavier seeds than DGN (1.25 g) and KDY (1.29 g). In 2023, differences among donors diminished, with similar weights (1.18–1.32 g). For stored pollen, weights ranged from 1.05 g (ZHI, 2021) to 1.48 g (DGN, 2020), with no significant differences in most cases, though DGN stored for two years (2020) produced heavier seeds (1.48 g). Reduced pollen stainability (59–68% after one year, 38–45% after two years) did not significantly affect seed weight, suggesting that a sufficient proportion of acetocarmine-positive grains remained to support normal seed development.

For seed length, fresh pollen in 2022 showed significant differences, with MJL (2.69 cm) and ZHI (2.66 cm) producing longer seeds than KDY (2.53 cm). In 2023, KDY yielded the shortest seeds (2.40 cm), while MJL maintained longer seeds (2.70 cm). For stored pollen, lengths were similar, ranging from 2.30 cm (ZHI, 2021) to 2.77 cm (MJL, 2021), with minimal differences among donors. These results indicate that reduced pollen stainability did not significantly affect seed length.

Seed diameter showed less variability, for example, in 2022, fresh ZHI pollen produced seeds with a larger diameter (0.93 cm) compared to KHY (0.81 cm); whereas in 2023, values were more uniform (0.82–0.87 cm). For stored pollen, diameters ranged from 0.79 cm (MJL, 2021) to 0.93 cm (ZHI, 2022), with no significant differences in most cases. This suggests that seed diameter is relatively insensitive to pollen donor or storage duration, even when pollen stainability decreased at 38–45% after two years.

**Figure 2:**
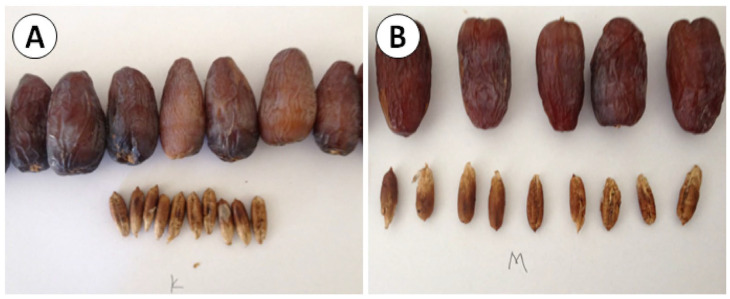
Effect of pollen source (xenia and metaxenia) on fruit and seed characteristics of ‘Mejhoul’ cultivar. (**A**) Shows fruits and seeds resulting from pollination with pollen donor from “KDY”. (**B**) corresponds to pollen donor “MJL”. Noticeable differences can be observed in fruit size, shape, and seed morphology, indicating the influence of paternal genotype on both fruit and seed development.

The Xenia phenomenon, where the pollen genotype affects seed characteristics, is evident in Table 5. In 2022, fresh MJL and ZHI pollen produced heavier and longer seeds, while DGN excelled with two-year-stored pollen. These differences suggest that the pollen donor influences embryo and endosperm development, likely through the expression of paternal genes regulating seed size [25] (Figure 2). The persistence of these effects with stored pollen indicates that biochemical components responsible for Xenia (e.g., phytohormones, epigenetic factors) remain functional despite reduced viability.

Swingle (1928) [25] was among the first to document Xenia in *Phoenix dactylifera*, observing that pollen from different cultivars affects seed size and weight. In this study, MJL and ZHI (fresh pollen, 2022) produced heavier and longer seeds, consistent with Nixon (1955), who reported that certain pollen cultivars induce larger seeds in the ‘Deglet Nour’ cultivar [13].

Our findings, based on acetocarmine staining, are consistent with the general trend observed in previous studies examining in vitro pollen germination and pollen tube growth at different storage temperatures [2,4,6,26,27]. Pollen viability was consistently higher at lower temperatures; however, the degree of preservation varied among the different cultivars analyzed. Mesnoua et al. (2018) [4], observed that date palm pollen stored at −20 °C retained up to 89% and at 4 °C 66% germination, after 13 months of storage for the cultivars Deglet Nour and Halwaya, respectively. Maryam et al. (2015) demonstrated that pollen stored after 12 months at −20 °C and −80 °C, had a higher percentage of in vitro germination with 27.4 and 32.24% respectively [27]. Likewise, the cryopreservation conservation method at −196 °C maintained high germination percentages (75–84%) without significant losses compared to fresh pollen, confirming it as the most effective long-term conservation method [6]. Anushma et al. (2018) [2] concluded that viability decreases dramatically at 4 °C, likely due to metabolic activity, whereas ultra-freezing or cryogenic storage effectively stabilizes pollen physiology. All these results corroborate our interpretation that refrigeration at 4 °C only ensures short-term cytological integrity, whereas sub-zero or cryogenic temperatures are necessary to preserve functional viability for several seasons.

These studies suggest that acetocarmine staining may overestimate viability compared to in vitro germination assays. Shaheen (2004) [26] evaluated 61 different fresh pollen sources, where viability ranged from 44.6 to 100% with acetocarmine staining, but only from 6 to 93% in the germination test, indicating that the staining reflects cytoplasmic integrity rather than the ability to germinate and elongate tubes. This result reveals that acetocarmine is useful for rapid testing, but does not discriminate between viable and functionally competent grains, whereas germination tests provide a more reliable indicator of fertility. Consequently, staining methods should be considered as preliminary indicators of cytological integrity.

Despite the promising results of pollen stored at temperatures above 4 °C, acquiring such preservation equipment is not feasible for most growers. Thus, the best alternative is to use a standard refrigerator at a constant 4 °C, with the recommendation to store pollen for the next season’s pollinations in sealed plastic or glass containers, preferably with a desiccant, to maintain dry conditions [28].

Finally, it is important to note that fruit set and seed are not direct indicators of pollen stainability, since many processes occur between pollen adhesion and fruit harvest that depend on female flower physiology. In this study, fruit set and seed were interpreted as complementary measures of the practical performance of stored pollen in the field.

## 3. Materials and Methods

### 3.1. Experimental Area Characterization

**Figure 3:**
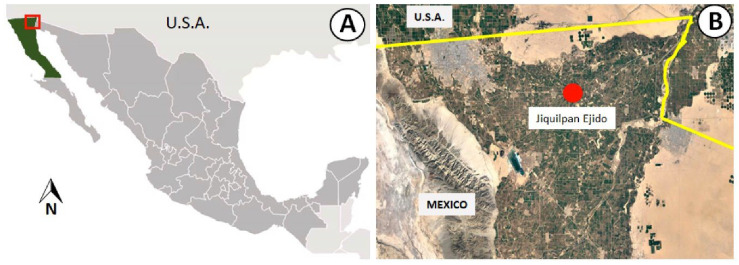
Geographic location of one of the main date production areas in Mexico. (**A**) The Mexicali Valley, an important region for commercial date palm cultivation in northwestern Mexico. (**B**) Detailed view showing the location of Ejido Jiquilpan within the Mexicali Valley (Cartographic data 2025 © Google).

The study was conducted during the 2022 and 2023 growing seasons in a 15-ha certified organic date palm orchard located in Ejido Jiquilpan (32°31′17″ N, 115°4′17″ W), in the Mexicali Valley, northwestern Mexico (Figure 3). The plants are spaced 8 × 8 m apart. The soil is classified as alluvial, derived from the dry bed of the Colorado River. Irrigation is applied via flooding, distributed in eight 15-cm cycles annually.

### 3.2. Phenotypic Characterization of Male Trees and Pollen Extraction

To identify the morphological characteristics of the four pollen sources in this study, standard descriptors defining the vegetative donor of date palms were obtained in January and February 2020 for the most common cultivars in the region: ‘Mejhoul’ (MJL), ‘Deglet Nour’ (DGN), ‘Khadrawy’ (KDY), and ‘Zahidi’ (ZHI) [29,30,31]. For morphological characterization, ten female plants of each cultivar were randomly selected, and three measurements were taken for each of nine vegetative characters per plant, as described in Table 6 (leaf length and width, middle leaflet length and width, middle spine length and width, spined portion length, and total leaflet and spine numbers per leaf).

Once characterized, twelve male date palms were selected as pollen sources, with phenotypic characteristics within the ranges of the four cultivars described above (Table 6). These male plants were grouped into four groups based on the phenotypic descriptions, as shown in Table 7. The male date palms, propagated from seeds, were at least 20 years old and free of diseases.

The pollen extraction method for the 2020–2023 seasons followed the technique used by local growers. All spathes were sequentially extracted from each tree as they matured. The spathes were taken to an outdoor shaded drying area, where they were removed from around the inflorescences. Each inflorescence was placed on a paper bed in a plastic tray to dry and release pollen grains.

The trays were covered with fine plastic mesh to allow air and natural light passage, while preventing contamination and protecting the inflorescences from bees. The inflorescences were maintained under these conditions for one to two weeks. To ensure uniform drying, one side of the inflorescence was exposed to natural light, and once the petals turned dark brown, the inflorescence was flipped to expose the other side. Inflorescences were placed in separate trays in groups of three to six, ensuring they belonged to the same phenotypic group.

The pollen was extracted manually; first, the pollen released by the inflorescences onto the paper bed was collected in a plastic container, and loose pollen was shaken from the dried inflorescence. Then, all flowers of the same donor were manually separated from the inflorescence and placed in the same container. Finally, the container’s contents were manually passed through a fine mesh to obtain clean, pure pollen, which was stored at 4 °C for one and two years in tightly sealed plastic containers.

### 3.3. Treatments and Experimental Design

Twelve treatments (T1–T12) were defined for this study, each with three replications (R1–R3). Four treatments used fresh pollen (T1–T4), four used one-year-stored pollen (T5–T8), and four used two-year-stored pollen (T9–T12), as shown in Table 8, sourced from the male palm donors defined in Table 7 (MJL, DGN, KDY, and ZHI).

Nine female ‘Mejhoul’ palms (P1–P9), aged 21 years with uniform vigor, planted in an 8 × 8 m pattern, were randomly selected. The first twelve bunches to emerge on each date palm were used for pollination in the 2022 and 2023 seasons.

A completely randomized design was employed, with each palm divided into four groups of three bunches (replications) pollinated with each of the four pollen sources (fresh, one-year-, and two-year-stored), as described in Table 9.

### 3.4. Pollination

Four groups of three bunches from each female plant (Table 9) were individually pollinated once using pollen from each male progenitor. Following the natural opening of female spathes, each was manually opened and covered with paper bags. Pollination was performed manually for all treatments using a latex bulb between the third and sixth day after spathe opening. Pollinated bunches were covered with paper bags to prevent contamination from other pollen sources, and the bags were removed two weeks post-pollination. To ensure maximum pollination efficiency, pollen was diluted with commercial wheat flour at a 1:1 ratio [3,17]. All female tree inflorescences were pollinated within a four-week period as they emerged and matured.

### 3.5. Pollen Stainability Assessment Method

Pollen stainability was evaluated in vitro using 1% acetocarmine staining. For the staining method, 0.001 g of pollen was weighed and added to 50 µL of distilled water, followed by 50 µL of 1% acetocarmine solution. The mixture was stirred for one minute and incubated at 4 °C for 30 min in the dark. Acetocarmine-positive grains, which absorbed the red dye, were considered to possess preserved cytoplasmic integrity, whereas acetocarmine-negative grains remained colorless [32,33]. In this study, the term “stainability” refers specifically to the percentage of acetocarmine-positive grains, used as an indirect indicator of pollen quality, while fruit set percentages were analyzed separately as measures of field effectiveness.

Pollen grains were counted using a Neubauer chamber, with 10 µL of the stained pollen suspension placed in the chamber. Counts were performed using an optical microscope (ZEISS Axioplan, Carl Zeiss AG, Oberkochen, Germany) to determine stainability percentages, calculated as the proportion of acetocarmine-positive grains relative to the total number of observed grains.

Three observations were taken for each pollen treatment from different areas of the slide, and the results were averaged to determine the maximum and minimum stainability values based on pollen age. These observations were conducted prior pollination in the 2022 and 2023 growing seasons. The percentage of acetocarmine-positive grains (stainability) was calculated using the following Equation [21]:

(1) $$Pollen\;Stainability\;(\%)=\frac{Total\;number\;of\;acetocarmine-positive\;pollen\;grains}{Total\;number\;of\;pollen\;grains\;observed}\times 100$$

### 3.6. Fruit Set Percentage

Fruit set percentages were calculated to evaluate the agronomic effectiveness of pollination with stored pollen, since fruit set reflects multiple processes beyond pollen stainability, including female flower physiology and post-fertilization development. In all female ‘Mejhoul’ plants, ten strands from each of the 12 bunches per plant were selected. The total number of fruit positions and set fruits per strand were counted to calculate the fruit set percentage (*FSP*) using the following Equation [17]:

(2) $$FSP=\frac{Total\;fruit\;set}{Total\;fruit\;positions}\times 100$$

**Figure 4:**
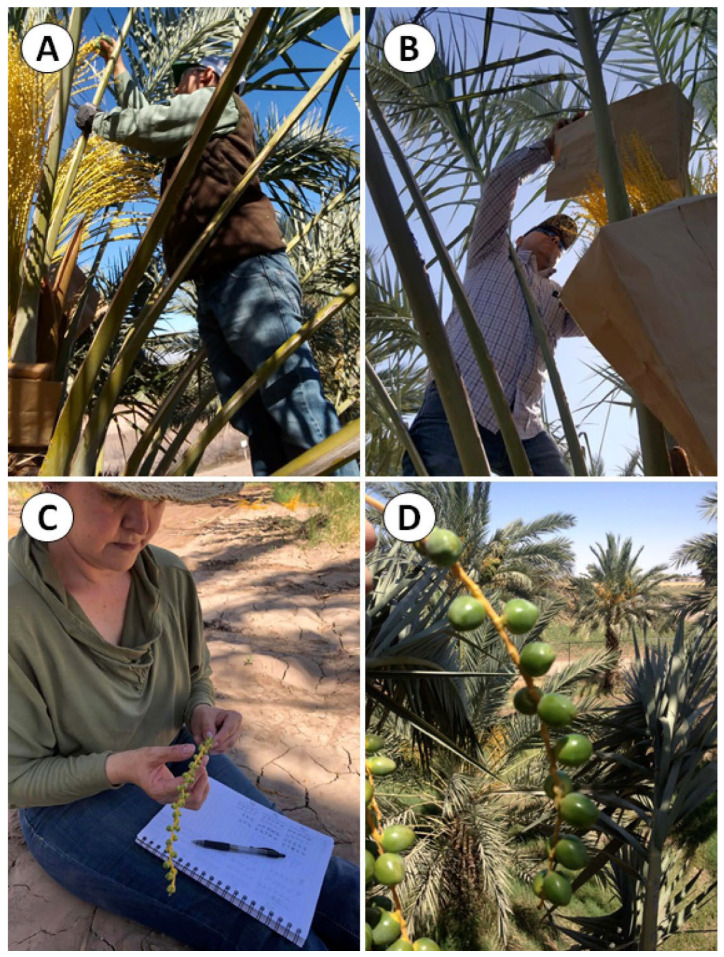
Stages of bunch management in female ‘Mejhoul’ date palm cultivar. (**A**) Manual pollination. (**B**) Covering of pollinated bunches. (**C**) Fruit set percentage calculation. (**D**) Fruit strand appearance after thinning.

After calculating the *FSP* for each bunch, strands were thinned to 12–14 dates per strand to achieve maximum fruit length in the ‘Mejhoul’ cultivar, following standard grower practices. Thinning was performed with sufficient spacing to allow fruit growth without competition for space (Figure 4).

### 3.7. Measurement of Physical Properties

Samples of fully mature fruits were collected, with ten fruits per bunch randomly selected during the harvest season (September–October 2022 and 2023). Fruits were individually weighed in grams using an analytical balance. Seeds were removed and weighed separately. Additional physical properties, such as fruit and seed length and diameter, were measured in centimeters.

### 3.8. Statistical Analysis

Data on fruit physical characteristics and fruit set percentages for each treatment across the two seasons were subjected to a one-way analysis of variance (ANOVA) in a balanced design. Cultivar means were compared using the least significant difference (LSD) test at a 5% significance level [34]. Variance analysis was performed using R Statistical Software version 3.5.0 [35,36]. Results were expressed as means ± standard deviation of three separate measurements per sample.

## 4. Conclusions

This study focused on staining-based assessment rather than germination assays, emphasizing the use of cytological integrity as a practical and rapid indicator, where acetocarmine staining was used to visually and quantitatively assess stainability in fresh and refrigerated pollen. The results demonstrated a progressive decline in stainability with increasing storage duration at 4 °C. However, pollen storage under these conditions maintained sufficient performance for effective pollination and satisfactory fruit set in the ‘Mejhoul’ cultivar, which does not require particularly high fruit set rates for commercial production.

The findings indicate that short- to medium-term pollen storage (up to one year) at 4 °C remains agronomically feasible for practical use, while longer storage periods result in a marked reduction in stainability and field performance. These results also reveal that pollen genotype influences fruit set and some fruit quality traits, supporting the existence of metaxenia effects and emphasizing the importance of selecting compatible and high-performing pollen sources for optimal yield and fruit quality.

The use of acetocarmine staining proved to be a practical and accessible method for assessing the integrity of date palm pollen. It offered a rapid and inexpensive way to analyze a large number of samples and distinguish pollen grains with intact cytoplasm. However, the results should be interpreted with caution, as this method does not directly measure functional viability. Its use in this study represents an important contribution due to its practical applicability in the field, as the technique can be easily replicated by date palm growers without the need for sophisticated equipment or advanced laboratory knowledge. This makes it a valuable and accessible tool for assessing pollen quality under real-world production conditions. However, future studies should include complementary assays such as in vitro germination, biochemical profiling, and molecular analyses to validate these findings and to refine long-term pollen storage strategies, including sub-zero or cryogenic conditions with strict humidity control.

## Figures and Tables

**Table 1:** Pollen stainability (%) by pollen donor and storage duration at 4 °C in 2022 and 2023.

Season	Pollen Donor	Fresh (2022/2023)	One-Year Stored (2021/2022)	Two-Year Stored (2020/2021)
2022	MJL	95.6 ± 1.9 a	68.3 ± 2.4 ab	45.4 ± 1.2 ab
	KDY	94.2 ± 2.0 a	63.1 ± 1.6 b	41.0 ± 1.4 b
	DGN	93.8 ± 1.8 a	66.0 ± 2.3 a	44.2 ± 2.1 a
	ZHI	91.2 ± 2.1 b	59.4 ± 1.5 c	38.8 ± 2.3 c
2023	MJL	95.0 ± 1.8 a	67.0 ± 1.3 ab	46.0 ± 2.1 ab
	KDY	94.0 ± 1.9 a	62.0 ± 2.5 b	40.0 ± 1.3 b
	DGN	95.0 ± 1.7 a	68.0 ± 2.2 a	45.0 ± 2.1 a
	ZHI	92.0 ± 2.0 b	60.0 ± 1.6 c	39.0 ± 1.7 c

Values are means ± SD of three measurements per pollen donor for each storage age. Means followed by the same letter are not significantly different at a 0.05 probability level. Stainability refers to the percentage of acetocarmine-positive pollen grains, reflecting cytoplasmic integrity rather than functional viability.

**Table 2:** Fruit set percentages in female ‘Mejhoul’ date palm cultivar during the 2022 and 2023 growing seasons using fresh and stored pollen (4 °C) for one and two years.

Pollen Donor	Fresh		One-Year Stored		Two-Year Stored	
	2022	2023	2021	2022	2020	2021
MJL	68.87 ± 11.14 bc	69.41 ± 7.64 b	58.22 ± 3.21 b	55.44 ± 3.98 b	45.20 ± 3.69 c	46.44 ± 3.98 ab
KDY	72.08 ± 13.23 b	64.71 ± 11.43 c	57.99 ± 5.79 b	54.52 ± 4.25 bc	50.53 ± 4.89 a	49.52 ± 4.25 a
DGN	81.71 ± 3.72 a	74.92 ± 10.93 a	64.50 ± 6.23 a	62.72 ± 4.32 a	52.02 ± 3.78 a	50.72 ± 4.32 a
ZHI	63.80 ± 12.74 c	65.15 ± 7.59 c	54.29 ± 4.59 c	53.60 ± 5.06 c	49.97 ± 5.98 b	49.60 ± 5.06 a

Values are means ± SD of 90 measurements per donor for each pollen age. Means followed by the same letter are not significantly different at *p* ≤ 0.05.

**Table 3:** Percentage reduction in fruit set between fresh pollen and pollen stored at 4 °C for one- and two-year periods during the 2022 and 2023 growing seasons.

Pollen Donor	One-Year Stored (%)			Two-Year Stored (%)		
	2021	2022	Average	2020	2021	Average
MJL	18.29	25.20	21.74	52.37	49.46	50.91
KDY	24.29	18.69	21.49	42.64	30.67	36.65
DGN	26.68	19.46	23.07	57.11	47.71	52.41
ZHI	17.52	21.55	19.53	57.67	31.35	44.51

Values represent the percentage reduction in fruit set compared to fresh pollen of the same season.

**Table 4:** Effect of pollen donor and storage duration (4 °C) on fruit weight, length, and diameter of ‘Mejhoul’ date palm cultivar during the 2022 and 2023 growing seasons.

Measuring Parameter	Pollen Donor	Fresh		One-Year Stored		Two-Year Stored	
		2022	2023	2021	2022	2020	2021
Weight (g)	MJL	18.05 ± 4.48 b	21.74 ± 2.80 ab	19.00 ± 2.91 a	20.96 ± 3.49 ab	20.35 ± 3.95 a	19.60 ± 2.89 a
	KDY	19.70 ± 1.9 ab	22.70 ± 3.21 a	20.49 ± 1.25 a	19.57 ± 2.66 b	20.35 ± 3.70 a	21.17 ± 2.94 a
	DGN	21.18 ± 4.49 a	17.70 ± 3.64 c	20.64 ± 2.68 a	22.47 ± 2.72 a	18.47 ± 3.37 a	20.93 ± 3.09 a
	ZHI	20.10 ± 2.33 ab	20.12 ± 2.60 b	19.45 ± 3.11 a	21.88 ± 2.30 a	20.01 ± 3.14 a	21.32 ± 4.4 a
Length (cm)	MJL	4.10 ± 0.53 c	4.58 ± 0.26 a	4.28 ± 0.33 a	4.42 ± 0.34 b	4.46 ± 0.39 a	5.00 ± 0.22 b
	KDY	4.27 ± 0.47 bc	4.55 ± 0.31 a	4.41 ± 0.39 a	4.26 ± 0.24 bc	4.46 ± 0.21 a	5.02 ± 0.22 ab
	DGN	4.49 ± 0.20 ab	4.16 ± 0.20 b	4.48 ± 0.30 a	4.77 ± 0.29 a	4.31 ± 0.28 a	5.17 ± 0.30 a
	ZHI	4.56 ± 0.28 a	4.69 ± 0.27 a	4.37 ± 0.42 a	4.19 ± 0.47 c	4.49 ± 0.36 a	4.97 ± 0.26 b
Diameter (cm)	MJL	2.42 ± 0.32 b	2.57 ± 0.12 a	2.58 ± 0.31 a	2.44 ± 0.24 b	2.63 ± 0.23 a	2.40 ± 0.19 a
	KDY	2.56 ± 0.24 ab	2.58 ± 0.24 a	2.51 ± 0.21 a	2.65 ± 0.28 a	2.51 ± 0.12 ab	2.55 ± 0.24 a
	DGN	2.62 ± 0.10 a	2.30 ± 0.25 b	2.67 ± 0.23 a	2.65 ± 0.22 a	2.59 ± 0.23 b	2.51 ± 0.20 a
	ZHI	2.69 ± 0.22 a	2.59 ± 0.13 a	2.62 ± 0.25 a	2.53 ± 0.30 ab	2.43 ± 0.27 a	2.56 ± 0.38 a

Values are means ± SD of 90 measurements per donor for each pollen age. Means followed by the same letter are not significantly different at *p* ≤ 0.05.

**Table 5:** Effect of pollen donor and storage duration (4 °C) on seed weight, length, and diameter of ‘Mejhoul’ date cultivar during the 2022 and 2023 growing seasons.

Measuring Parameter	Pollen Donor	Fresh		One-Year Stored		Two-Year Stored	
		2022	2023	2021	2022	2020	2021
Weight (g)	MJL	1.47 ± 0.14 a	1.30 ± 0.24 a	1.43 ± 0.15 a	1.42 ± 0.22 a	1.27 ± 0.16 b	1.15 ± 0.14 bc
	KDY	1.29 ± 0.12 bc	1.18 ± 0.11 a	1.41 ± 0.23 a	1.33 ± 0.16 a	1.35 ± 0.16 ab	1.24 ± 0.17 ab
	DGN	1.25 ± 0.21 c	1.30 ± 0.17 a	1.40 ± 0.12 a	1.36 ± 0.15 a	1.48 ± 0.20 a	1.37 ± 0.21 a
	ZHI	1.43 ± 0.11 ab	1.32 ± 0.14 a	1.30 ± 0.14 a	1.37 ± 0.15 a	1.41 ± 0.19 ab	1.05 ± 0.16 c
Length (cm)	MJL	2.69 ± 0.13 a	2.70 ± 0.15 a	2.77 ± 0.12 a	2.66 ± 0.15 a	2.55 ± 0.21 bc	2.44 ± 0.11 ab
	KDY	2.53 ± 0.16 b	2.40 ± 0.13 c	2.68 ± 0.15 ab	2.71 ± 0.15 a	2.51 ± 0.20 c	2.50 ± 0.17 a
	DGN	2.60 ± 0.13 ab	2.52 ± 0.15 bc	2.64 ± 0.13 ab	2.66 ± 0.15 a	2.73 ± 0.22 a	2.59 ± 0.20 a
	ZHI	2.66 ± 0.17 ab	2.54 ± 0.14 b	2.60 ± 0.20 b	2.70 ± 0.13 a	2.71 ± 0.11 ab	2.30 ± 0.19 b
Diameter (cm)	MJL	0.88 ± 0.04 ab	0.82 ± 0.05 a	0.90 ± 0.06 a	0.89 ± 0.05 a	0.88 ± 0.04 a	0.79 ± 0.03 b
	KDY	0.81 ± 0.04 c	0.82 ± 0.07 a	0.85 ± 0.07 a	0.91 ± 0.05 a	0.91 ± 0.04 a	0.83 ± 0.04 ab
	DGN	0.83 ± 0.08 bc	0.85 ± 0.06 a	0.88 ± 0.03 a	0.89 ± 0.06 a	0.91 ± 0.04 a	0.85 ± 0.06 a
	ZHI	0.93 ± 0.04 a	0.87 ± 0.03 a	0.87 ± 0.04 a	0.93 ± 0.07 a	0.91 ± 0.06 a	0.81 ± 0.05 ab

Values are means ± SD of 90 measurements per donor for each pollen age. Means followed by the same letter are not significantly different at *p* ≤ 0.05.

**Table 6:** Morphological characterization ranges for four female date palm cultivars grown in Mexico.

Cultivar	Leaf Length (cm)	Leaf Width (cm)	Middle Leaflet Length (cm)	Middle Leaflet Width (cm)	Middle Spine Length (cm)	Middle Spine Width (cm)	Spined Portion Length (cm)	Leaflet Number	Spine Number
MJL	304–376	65–82	50–64	2.8–3.4	11–16.5	0.5–0.8	57–88	143–156	20–30
DGN	327–400	63–74	49–53	2.9–3.7	8.1–13.5	0.6–0.8	103–122	117–182	36–44
KDY	316–329	71–89	47–57	3.6–4.5	9.8–13.2	0.5–0.6	57–64	130–143	20–22
ZHI	443–468	57–64	46–52	2.7–3.7	5.2–7.6	0.6–0.7	90–120	117–133	28–32

Values correspond to adult female plants located in the Jiquilpan Ejido (32°31′17″ N, 115°4′17″ W), Mexicali Valley, Mexico.

**Table 7:** Phenotypic and morphological traits of male date palm seedlings selected for pollen extraction during the 2020–2023 seasons.

Palm ID	Phenotypic Similarity	Leaf Length (cm)	Leaf Width (cm)	Middle Leaflet Length (cm)	Middle Leaflet Width (cm)	Middle Spine Length (cm)	Middle Spine Width (cm)	Spined Portion Length (cm)	Leaflet Number	Spine Number
P1	MJL	308	72	52	3.4	13.7	0.7	61	147	24
P2	MJL	348	71	55	3.2	12.4	0.5	73	156	26
P3	MJL	354	80	63	3.2	12.3	0.5	66	152	21
P4	MJL	337	76	57	3.3	12.8	0.6	69	150	29
P5	DGN	330	66	49	3.1	10.3	0.6	104	169	42
P6	DGN	397	69	50	3.1	9.1	0.7	105	180	37
P7	DGN	385	71	53	3.5	12.1	0.7	112	155	38
P8	KDY	323	82	51	4.0	11.2	0.5	62	137	21
P9	KDY	321	79	56	3.7	11.9	0.6	60	140	20
P10	KDU	326	87	47	4.2	10.2	0.5	63	130	22
P11	ZHI	450	61	48	3.1	6.7	0.6	108	120	30
P12	ZHI	447	60	46	2.9	7.5	0.6	115	127	32

Male plants located in the Jiquilpan Ejido (32°31′17″ N, 115°4′17″ W), Mexicali Valley, Mexico.

**Table 8:** Description of fresh and stored pollen sources (4 °C) used for pollination treatments during the 2022 and 2023 growing seasons.

Treatments	Replications	Pollen Donor	Pollen Age	Extraction Year	Pollination Season
T1–T4	R1–R3	MJL, DGN, KDY, ZHI	Fresh	2022 2023	2022 2023
T5–T8	R1–R3	MJL, DGN, KDY, ZHI	one-year-stored	2021 2022	2022 2023
T9–T12	R1–R3	MJL, DGN, KDY, ZHI	Two-years stored	2020 2021	2022 2023

**Table 9:** Distribution of female ‘Mejhoul’ date palms selected for pollination treatments according to pollen age.

Female Palms	Treatments	Replications	Pollen Age
P1–P3	T1–T4	R1–R3	Fresh
P4–P6	T5–T8	R1–R3	One-year-stored
P7–P9	T9–T12	R1–R3	Two years stored

Each treatment was applied to three female palms with three replications per pollen donor.

## Data Availability

The original contributions presented in this study are included in the article. Further inquiries can be directed to the corresponding authors.

## Abbreviations

The following abbreviations are used in this manuscript:

MJL	Mejhoul
KDY	Khadrawy
DGN	Deglet Nour
ZHI	Zahidi
